# Early Life Stress in Depressive Patients: HPA Axis Response to GR and MR Agonist

**DOI:** 10.3389/fpsyt.2014.00002

**Published:** 2014-01-24

**Authors:** Cristiane von Werne Baes, Camila Maria Severi Martins, Sandra Márcia de Carvalho Tofoli, Mário Francisco Juruena

**Affiliations:** ^1^Stress and Affective Disorders – SAD Program, Mental Health Graduate Program, Department of Neuroscience and Behaviour, Faculty of Medicine of Ribeirão Preto, University of São Paulo, São Paulo, Brazil; ^2^Institute of Psychiatry, King’s College London, London, UK

**Keywords:** early life stress, hypothalamic-pituitary-adrenal, cortisol, glucocorticoid receptors, mineralocorticoid receptors, depression

## Abstract

**Background:** Evidence indicates that early life stress (ELS) can induce persistent changes in the hypothalamic-pituitary-adrenal (HPA) axis to respond to stress in the adult life that leads to depression. These appear to be related to the impairment of HPA hormones through binding to glucocorticoid (GR) and mineralocorticoid receptors (MR). The aim of this study was to evaluate the impact of ELS in HPA axis response to challenges with GR and MR agonists in depressed patients.

**Methods:** We included 30 subjects, 20 patients with current major depression (HAM-D_21_ ≥ 17). Patients were recruited into two groups according to ELS history assessed by the Childhood Trauma Questionnaire (CTQ). The cortisol measures in the saliva and plasma were evaluated after using (at 10:00 p.m.) placebo, fludrocortisone (MR agonist), or dexamethasone (GR agonist).

**Results:** Depressed patients showed a significantly lower salivary cortisol upon waking after placebo compared with controls. Moreover, cortisol awakening responses (CAR) after MR agonist were found to be lower in depressed patients than in controls. With CTQ scores, HAM-D_21_, body mass index and CAR after placebo, GR agonist, MR agonist we found in a Linear Regression model that depressive patients with ELS (*p* = 0.028) show differences between placebo vs. MR agonist (*R* = 0.51; *p* < 0.05) but not after GR agonist; in depressive patients, without ELS the data show differences between placebo vs. MR agonist (*R* = 0.69; *p* < 0.05); but now as well placebo vs. GR agonist (*R* = 0.53; *p* < 0.05).

**Conclusion:** Our findings indicate that MR activity is impaired in depressed patients compared with controls. Furthermore, in spite of the previous limitations described, in depressed patients with ELS, there was suppression by MR agonist, indicating that patients with ELS are sensitive to MR agonists. In contrast with depressed patients without ELS, we find suppression after both MR and GR agonist. These data suggested that in ELS an imbalance exists between MR and GR with MR dysfunction.

## Introduction

Stressful life events play an important role in the pathogenesis of depressive disorders and are well established as acute triggers of psychiatric illness (1). According to the literature, early life stress (ELS), such as child abuse, neglect, or parental loss, has been associated with significant increase in the risk of developing depression in adulthood (2–5). Recent studies show that ELS can also influence the clinical course and a poorer treatment outcome of depression (6, 7). Child abuse and neglect can be perceived as agents for neurodevelopment disturbance and, depending on when it occurs, can cause neurological “scars” in some structures, which could make some individuals vulnerable to certain types of psychopathology, especially depression (4, 8, 9).

Considerable evidence suggests that this vulnerability for developing psychiatric disorders is associated with changes in neurobiological systems related to stress regulation. Abnormalities in hypothalamic-pituitary-adrenal (HPA) axis have been widely described in the literature, in people experiencing mood disorder (10–12). Moreover, studies indicate that stress in early phases of development can induce persistent changes in the ability of the HPA axis to respond to stress in the adult life, and that mechanism can lead to a raised susceptibility to depression (13, 14). However, despite strong evidence in the literature suggesting that ELS is associated with abnormalities in HPA axis that leads to depression, there is no clear consensus whether the ELS leads to hyper- or hypo-activation of this axis (15).

In this sense, one aspect of the function of HPA axis that recently received particular attention for the understanding of HPA axis disturbances is the measurement of salivary cortisol in response to awakening (16–18). The cortisol awakening responses (CAR) are the rapid increase in cortisol levels that peaks approximately 30–45 min after awakening in the morning (16, 18). CAR is considered a reliable measure of basal HPA axis activity and represents the acute response of the HPA axis to awakening (19). Although in recent decades this phenomenon has been studied mainly in healthy populations, recently, some studies have described altered CAR in psychiatric disorders, such as depression (20–24). However, the findings related to depression and CAR are heterogeneous. While some studies found an increased CAR in depressed patients (21, 24), other studies have reported a blunted CAR in depression (22). In addition, some studies have demonstrated that increased CAR can be an important risk factor for the development of depression in adults (25, 26).

One of the mechanisms thought to be involved in these abnormalities is the impaired feedback inhibition of the HPA axis by the circulating glucocorticoids (27). Hypothalamic-pituitary activity leads to the production of glucocorticoids from the adrenal cortex. In turn, glucocorticoids mediate their actions, including a feedback inhibition, through two distinct intracellular receptor subtypes: the type I or mineralocorticoid receptor (MR) and the type II or glucocorticoid receptor (GR). These receptors differ in their affinity for glucocorticoids, with MR demonstrating the highest affinity for cortisol and GR demonstrating lowest affinity for cortisol (28–31).

Thus, the dysfunction of MR and GR has been implicated in stress-related psychiatric diseases such as depression (32–36). In this sense, several studies have been published since the 70s with dexamethasone suppression test (DST), a synthetic glucocorticoid that binds preferentially to GR (15, 37–40). Most studies have demonstrated that severely depressed patients often show non-suppression and impaired feedback inhibition by dexamethasone, which is indicative for dysfunction of corticosteroid receptors, especially GR (34, 41–43). However, due to low sensitivity of the DST (20–50%) to distinguish between patients with major depression and patients with other psychiatric disorders or healthy subjects (39, 44), Holsboer et al. have developed a more sensitive neuroendocrine test (45, 46) that combines the DST and the corticotropin-releasing hormone (CRH) stimulation test, and it is called the dexamethasone/corticotrophin-releasing hormone (Dex/CRH) challenge test. A suppressive test using another synthetic glucocorticoid, prednisolone, has recently been developed. Current evidences suggest that the prednisolone suppression test (PST), in contrast to the DST and the Dex/CRH test, probes both the MR and the GR and hence provides a more valid test of the HPA axis in depression (33, 34, 40, 47).

On the other hand, some studies have been published using challenges that assess, preferentially, MR function in depression using fludrocortisone (MR agonist) or spironolactone (MR antagonist), but these studies are still restricted and revealed unclear results. In this sense, Buckley et al. (48) evaluated the acute effects of fludrocortisone (0.5 mg) on nocturnal HPA axis activity in healthy subjects, finding that it is able to inhibit nocturnal activity of the HPA axis, showing significant clinical implications for the treatment of insomnia and depression (48). In a recent study, Lembke et al. (49) reported that patients with psychotic major depression (PMD) have diminished feedback inhibition of HPA axis in response to fludrocortisone compared to healthy control subjects (49). Otte et al. (50) examined the role of MR in the response to antidepressants through stimulation and blockade of MR and found decreased plasma cortisol levels in depressed patients treated with fludrocortisone as adjunct to escitalopram and the stimulation of MR with fludrocortisone accelerated the treatment response. Furthermore, the combination of spironolactone and escitalopram increased plasma cortisol levels during treatment (50). Still regarding the evaluation of blockade of MR with spironolactone, both studies by Heuser et al. (51) in healthy controls and Young et al. (52) in depressive patients showed a significant increase in cortisol levels in subjects treated with spironolactone.

Therefore, these data have lead to the hypothesis that an imbalance in MR and GR functioning may be a risk factor for depression (29). Moreover, results from studies examining the relationship between ELS and HPA axis indicate that ELS, in combination with the genetic background, seems to sensitize certain circuits in the brain and leads to persistent alterations in reactivity and sensitization of the HPA axis to subsequent stress, as reflected in an altered MR/GR balance that contributes to the risk for depression (53–56). In this area, the majority of studies is restricted to assessment of GR by the traditional DST and Dex/CRH test (8, 24, 55, 57–59) and only a few recent studies used the PST that assesses both GR and MR (34, 36, 47, 60). Moreover, according to our knowledge to date, no studies have been published that specifically evaluate the functioning of MR in depressive patients with ELS with neuroendocrine challenges.

Finally, regarding the assessment of MR and GR receptors through the CAR in depressed patients, studies are still restricted and as well as other studies in this area are limited to assessment of GR with DST (61). Thus, more studies are needed, with tests that assess both GR and MR receptors through the CAR, for a better understanding of the role of the ELS on MR function in depressive patients. Therefore, we hypothesize that the ELS results in a persistent dysfunction of HPA axis and GR/MR receptors, leading to MR malfunction, in adult depressive patients. Based on these data, in the present study, we evaluated the impact of ELS in HPA axis response to challenges with GR and MR agonist in depressed patients.

## Materials and Methods

### Study design

The study used a single-blind, non-randomized, placebo-controlled, repeated-measure design. Before each study day, the subjects were instructed to take one capsule (at 10:00 p.m.), containing placebo, dexamethasone (0.5 mg), or fludrocortisone (0.5 mg). No alcohol, coffee, tea, or meals were allowed after each capsule. Salivary samples were collected at 10:00 p.m. right after drug administration, the following day immediately upon awakening, 30 min later, 60 min later, and before plasma collection at 9:00 a.m.

The study protocols were all approved by the Research Ethics Committee of the General Clinical Hospital, Faculty of Medicine of Ribeirao Preto, University of Sao Paulo.

### Participants

A total of 30 subjects, aged 18–65 years, including 20 depressed patients and 10 healthy controls participated in the study. Written informed consent was obtained from all subjects.

We examined depressed inpatients at the Day Hospital Unit of the General Clinical Hospital. Patients were included in this study if they had a diagnosis of depressive episode according to DSM-IV criteria (62) and a score of 17 or more in the Hamilton Depression Rating Scale [HAM-D_21_; (63)]. For convenient reasons it was not possible to test the patients in a drug-free state. All 20 patients were taking medication during the assessment. Thirteen patients were taking benzodiazepines (diazepam and clonazepam); 12 SSRIs (fluoxetine and sertraline); 9 antipsychotics (chlorpromazine, haloperidol, risperidone, quetiapine, and olanzapine); 6 tricyclics (imipramine, amitriptyline, clomipramine, and nortriptyline); 6 other antidepressants (bupropion and venlafaxine); 4 mood stabilizers (lithium, lamotrigine, topiramate, and oxcarbazepine); and 3 other drugs (promethazine). Exclusion criteria for the patient group were a history of hypersensitivity to corticosteroids or steroid use, heavy smoking (more than 25 cigarettes a day), a viral illness during the preceding 2 weeks, pregnancy or lactation, alcohol dependence, and significant physical illness (severe allergy, autoimmune disease, hypertension, malignancy, or hematological, endocrine, pulmonary, renal, hepatic, gastrointestinal, or neurological disease). We also excluded patients with current alcohol or drug abuse/dependence, mental retardation, psychotic symptoms unrelated to their depressive disorder, or an organic cause for their depression.

On the basis of positive history of ELS, depressed patients were divided into two groups. The first included those with ELS and the second included those without ELS. We included in the group with ELS only the patients with scores moderate to severe or severe to extreme according to the Childhood Trauma Questionnaire [CTQ; (64)]. Among the 20 depressed patients evaluated, 13 (65%) had experienced some form of ELS and 7 (35%) had no history of ELS.

The healthy controls, matched as a group to the depressed patients according to gender and body mass index (BMI; within a range of ±5 kg/m^2^), were recruited from hospital staff, students, and the local community via public advertisement. The control group participants were physically healthy on the basis of a complete medical history and examination, were not taking any psychotropic medication, were not taking any hormonal medication (including oral contraceptives), and had no history of hypersensitivity to corticosteroids. Healthy individuals were excluded if they had a personal history or first-degree relative history of a DSM-IV axis I disorder or history of ELS.

### Clinical assessments

Demographic, clinical, and psychosocial data were obtained from medical charts and semi-structured clinical interviews carried out by researchers. All subjects were interviewed by a psychiatrist using the Mini International Neuropsychiatric Interview [MINI; (65)], version in Portuguese translated and adapted by Amorim (66) for confirmation of the diagnosis of major depression. The MINI is a brief structured interview designed to assess criteria for the major axis I psychiatric disorders classified in DSM-IV and ICD-10. The diagnostic assessment was conducted using the MINI for DSM-IV diagnoses by two seniors psychiatrists (Mário Francisco Juruena; Cristiane von Werne Baes) trained and certified to the use of the standardized interviews. The interviewers had long-standing experience in the administration of standardized interviews. For assessment of the severity of depression, participants were interviewed using the 21-item Hamilton Depression Rating Scale [HAM-D_21_; (63)]. Patients were required to have a score of at least 17 on the 21-item HAM-D for inclusion in this study. We used a cut-off score of 17 or more in order to define a sufficient level of depression ensuring the inclusion of patients with moderate to severe clinical levels of depressive illness. The basis for this was that this is a score generally used in treatment trials in depression, and specifically equates to that used in STAR*D, which used a 17-item HAM-D cut-off of 14, which is equivalent to a cut-off of 16 on the 21-item HAM-D (67). However, there is no consensus in the literature concerning a specific cut-off point defining mild to moderate depression in the 21-item HAM-D.

#### Early life stress measures

The ELS was assessed using the CTQ (64). The CTQ is a retrospective self-report questionnaire that investigates history of abuse and neglect during childhood and can be applied to adolescents (from 12 years) and adults where the responder assigns values of frequency to 28 graduate assertive issues related to situations arising in childhood. The CTQ evaluates five subtypes of ELS:

Emotional abuse: verbal assaults on a child’s sense of worth or well-being or any humiliating or demeaning behavior directed toward a child by an adult or older person;
Physical abuse: bodily assaults on a child by an adult or older person that posed a risk of or resulted in injury;
Sexual abuse: sexual contact or conduct between a child younger than 18 years of age and an adult or older person;
Emotional neglect: the failure of caretakers to meet children’s basic emotional and psychological needs, including love, belonging, nurturance, and support;
Physical neglect: the failure of caretakers to provide for a child’s basic physical needs, including food, shelter, clothing, safety, and health care (poor parental supervision was also included in this definition if it placed children’s safety in jeopardy) (68).

The items are rated on a Likert scale ranging from 1 (never) to 5 (very often). Furthermore, the scores range from 5 to 25 for each type of ELS. The instrument also contains a subscale of minimization/denial to identify individuals responding in a socially desirable manner, and a cut point for ELS was defined as when one of these experiences before the age of 18 reached a degree of at least moderate to severe, or severe to extreme according to classification of CTQ. The version in Portuguese was translated and adapted by Grassi-Oliveira et al. (69).

### Endocrine assessments

The suppression tests were administered shortly after study admission for patients and controls (range 5–10 days). On day 1, the subjects were instructed to take one capsule (at 10:00 p.m.) containing placebo, followed by assessment of cortisol. Forty-eight hours after the administration of placebo (day 4) subjects took the second capsule (at 10:00 p.m.) containing fludrocortisone 0.5 mg and they repeated the assessment of cortisol. On day 7, 48 h after the administration of fludrocortisone, they took the third capsule (at 10:00 p.m.) containing dexamethasone 0.5 mg and repeated the assessment of cortisol.

Cortisol assessment consists of analysis of five salivary samples and one plasma sample. Salivary samples were collected using Salivettes (Sarstedt, Germany) that contained an untreated cotton swab. Subjects were instructed to collect the first Salivette at 10:00 p.m. after drug administration and not to drink alcohol, exercise, or engage in stressful activities right after drug intake. Participants were also instructed not to smoke, drink caffeine, eat, or brush their teeth in the 60 min prior to salivary collection. This instruction was given verbally accompanying the sampling tubes. New salivary samples were collected immediately upon awakening, 30 and 60 min later; these three samples were used to determine the CAR. Another salivary sample was collected at 9:00 a.m. before collection of plasma cortisol. Blood and salivary samples were immediately centrifuged at 3000*g* for 10 min, aliquoted, and stored at −40°C and analyzed at the Endocrinology Laboratory of the General Clinical Hospital, Faculty of Medicine of Ribeirao Preto, University of Sao Paulo, by RIA (70). Detection limits and the intra-assay and inter-assay coefficients of variation were: 1.68 nmol/L, 2.1 and 9.3% for salivary cortisol and 33.10 nmol/L, 2.8 and 10.4% for plasma cortisol.

### Statistical analysis

**Figure 1:**
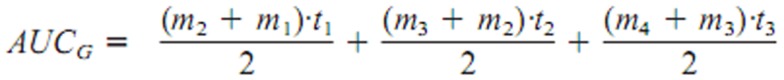
**Formula for calculation of area under the curve with respect to ground (AUCg)**. Adapted from Pruessner et al. (71). Note: *m* denotes single measurements; *t* denotes time interval between measurements.

All values are presented as mean and standard error of the mean. The main parameter of CAR used in this study was the area under the curve with respect to ground (AUCg; nmol × h/L). AUCg was calculated according to the trapezoidal method described by Pruessner et al. (71), which considers the distance of individual measurements to the baseline and represents an estimate of total cortisol secretion within the first hour after awakening, as demonstrated below (Figure 1):

Chi-squared tests with Bonferroni corrected *post hoc* tests were used to assess the significance for dichotomous variables. Continuous variables were calculated by *t*-tests to compare differences between depressed patients and control group and by one-way analysis of variance (ANOVA) with Tukey corrected *post hoc* tests for comparisons among patients with ELS, without ELS, and controls. We used a general linear model (GLM) analysis for repeated measures to examine both between-group differences (depressed patients with ELS vs. without ELS vs. controls) and within-group differences (placebo vs. fludrocortisone vs. dexamethasone) in salivary cortisol at 10:00 p.m., immediately upon awakening, 30 and 60 min later and 9:00 a.m. Further analyses were conducted using one-way ANOVA with Tukey corrected *post hoc* tests for a comparison of mean CAR levels between placebo vs. fludrocortisone vs. dexamethasone. Pearson’s test was used to examine the correlations between CTQ scores and plasma cortisol.

The main objective of the present study was to evaluate the impact of the severity of CTQ on, MR and GR in depressive patients with and without ELS for which a multiple regression analysis was conducted. Therefore, we have controlled some measures as depression scores (HAM-D), and BMI and use CTQ scores as continuous measures correlating with CAR AUC (nmol × h/L) after placebo vs. fludrocortisone vs. dexamethasone. All analyses were conducted using the Statistical Package for the Social Sciences, SPSS for Windows, release 15.0. A value of *p* < 0.05 was considered statistically significant.

## Results

### Clinical assessments

Depressed patients and controls did not differ significantly in gender (*X*^2^ = 1.87; df = 1.0; *p* = 0.17) and BMI (*t* = 0.47; df = 26.0; *p* = 0.64). Patients were significantly older than controls (*t* = 3.2; df = 27.02; *p* = 0.003), mean age was 38.8 (±2.2) years in patients and 29.4 (±1.8) years in controls. Among the patients, 11 (55%) had a personality disorder, 17 (85%) had attempted suicide, and 18 (90%) had a positive family history of psychiatric disorders. According to CTQ, more than half of the patient group (13/20; 65%) had experienced some subtype of ELS: specifically, 11 had experienced emotional abuse, 10 reported physical neglect, 9 reported emotional neglect, 9 had experienced physical abuse, and 7 had experienced sexual abuse. Furthermore, regarding the amount of subtypes experienced by patients with ELS, most of them (92.4%) reported having experienced two to five subtypes of ELS. By definition, depressed patients with ELS had higher mean CTQ scores than depressed patients without ELS (*p* < 0.001) and controls (*p* < 0.001), mean CTQ total score was 74.0 (±5.1) in depressed patients with ELS, 38.1 (±1.0) in depressed patients without ELS, and 29.6 (±2.0) in controls. There were no differences in mean CTQ scores between depressed patients without ELS and controls (*p* = 0.38). In addition, patients with ELS had significantly higher scores than depressed patients without ELS and controls in all CTQ subscales of abuse and neglect. There were no differences between patients groups with or without ELS in gender, age, and BMI. The groups differed significantly regarding the diagnosis of axis I psychiatric disorders (*X*^2^ = 4.12; df = 1.0; *p* = 0.04). In the group of patients with ELS 100% (13/13) of the sample had unipolar depression; on the other hand, in the group without ELS almost 30% (2/7) of the sample had bipolar depression. Patients with or without ELS showed no significant differences in the other demographic and clinical variables (Table 1).

**Table 1:** **Demographic and clinical features of depressed patients with or without early life stress**.

	With early life stress *n* = 13 (65%)	Without early life stress *n* = 7 (35%)	*P*
Gender, *n* (%)			0.79
Female	10 (76.9)	5 (71.4)	
Male	3 (23.1)	2 (28.6)	
Age, years (±SEM)	39.5 (±2.7)	37.4 (±4.3)	0.67
BMI, Kg/m^2^(±SEM)	29.2 (±2.0)	25.4 (±2.3)	0.24
Ethnicity, *n* (%)			0.59
Caucasian/white	7 (53.8)	5 (71.4)	
Mulatto/mixed race	3 (23.1)	2 (28.6)	
Black	2 (15.4)	0 (0)	
Asian	1 (7.7)	0 (0)	
Education, *n* (%)			0.68
≤4 years	2(15.4)	2 (28.6)	
5–8 years	1 (7.7)	1 (14.3)	
9–11 years	5 (38.5)	1 (14.3)	
≥11 years	5 (38.5)	3 (42.9)	
Marital status, *n* (%)			0.40
Never-married	4 (30.8)	2 (28.6)	
Married	7 (53.8)	4 (57.1)	
Separated/divorced	2 (15.4)	1 (14.3)	
Employment status, *n* (%)			0,64
Employed	1 (7.7)	1 (14.3)	
Unemployed	12 (92.3)	6 (85.7)	
Smokers, *n* (%)	4 (30.8)	2 (28.6)	0.92
Clinical disease, *n* (%)	6 (46.2)	4 (57.1)	0.64
Axis I psychiatric disorders, *n* (%)			0.04
Unipolar depression	13 (100)	5 (71.4)	
Bipolar depression	0 (0)	2 (28.6)	
Axis II psychiatric disorders, *n* (%)	8 (61.5)	3 (42.9)	0.42
Positive family history, *n* (%)	12 (92.3)	6 (85.7)	0.64
Suicide attempts in the past, *n* (%)	12 (92.3)	5 (71.4)	0.21
CTQ, total score (±SEM)	74.0 (±5.1)	38.1 (±1.0)	<0.001
Emotional abuse	18.1 (±1.5)	9.7 (±0.7)	<0.001
Physical abuse	14.2 (±1.6)	6.1 (±0.6)	<0.001
Sexual abuse	11.5 (±2.1)	5.1 (±0.1)	0.01
Emotional neglect	17.0 (±1.3)	11.0 (±1.1)	<0.01
Physical neglect	13.4 (±1.2)	6.1 (±0.5)	<0.001
HAM-D_21_ score (±SEM)	28.6 (±1.5)	25.2 (±1.9)	0.20

*SEM, standard error of mean; NS, non-significant; CTQ, Childhood Trauma Questionnaire; HAM-D_21_, Hamilton Depression Rating Scale*.

### Endocrine assessments

When comparing depressed patients vs. healthy controls, patients showed a significantly lower salivary cortisol than control subjects upon waking after placebo (*t* = −2.2; df = 28.0; *p* = 0.03). Mean cortisol upon waking was 23.6 (±3.6) nmol/L in patients and 36.3 (±3.7) nmol/L in controls.

**Figure 2:**
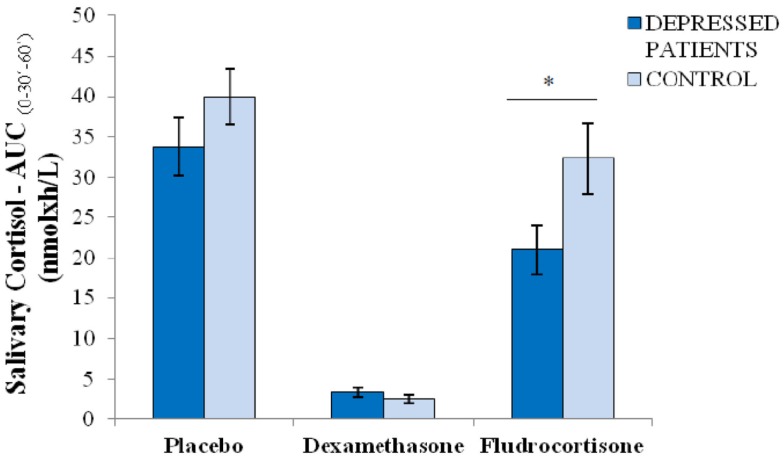
Cortisol awakening response (measured as area under the curve) after placebo, dexamethasone (GR agonist), and fludrocortisone (MR agonist) in 20 depressed patients and 10 healthy controls; *p < 0.05. Note: AUCg_(0–30′–60′)_ = Area under the curve from salivary cortisol immediately upon awakening, 30 and 60 min later (nmol × h/L); values are means, with standard errors represented by vertical bars.

Moreover, we calculated the CAR, measured using the AUCg_(0–30′–60′)_. Depressed patients and controls did not differ significantly in the CAR both after placebo (33.7 ± 3.6 vs. 40.0 ± 3.9 nmol × h/L; *p* = 0.36) and after dexamethasone (3.4 ± 0.6 vs. 2.5 ± 0.5 nmol × h/L; *p* = 0.39). However, depressed patients showed a significantly lower CAR after fludrocortisone compared with controls (21.0 ± 3.1 vs. 32.3 ± 4.4 nmol × h/L; *p* = 0.04). In summary, these results showed that depressed patients have higher suppression by fludrocortisone, a MR agonist, but a similar suppression by dexamethasone, a GR agonist, compared to controls (Figure 2).

When comparing depressed patients with ELS vs. without ELS vs. controls, the GLM analysis did not show a main effect of groups (*F* = 1.31; df = 2.0; *p* = 0.28), nor a group × time interaction (*F* = 0.79; df = 8.0; *p* = 0.61), but showed a main effect of time (*F* = 19.5; df = 4.0; *p* < 0.001). The GLM analysis also showed a main effect of challenge (*F* = 92.8; df = 2.0; *p* < 0.001) and a challenge × time interaction (*F* = 14.4; df = 8.0; *p* < 0.001). Subsequent pairwise analysis indicated that there was a difference between placebo and fludrocortisone in their effects on salivary cortisol (*p* = 0.002), between placebo and dexamethasone (*p* < 0.001), and between fludrocortisone and dexamethasone (*p* < 0.001). The GLM analysis did not show a significant group × challenge interaction (*F* = 1.88; df = 4.0; *p* = 0.12) and a group × challenge × time interaction (*F* = 1.09; df = 16.0; *p* = 0.39).

**Figure 3:**
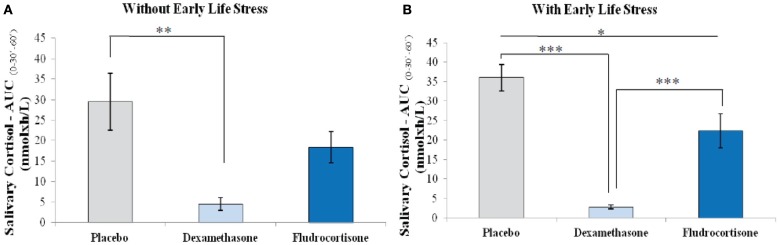
Cortisol awakening response (measured as area under the curve) after placebo, dexamethasone (GR agonist), and fludrocortisone (MR agonist) in (A) depressed patients without early life stress (*n* = 7); placebo vs. dexamethasone **p < 0.01 and (B) depressed patients with early life stress (*n* = 13); placebo vs. fludrocortisone *p = 0.02; placebo vs. dexamethasone, and dexamethasone vs. fludrocortisone ***p ≤ 0.001. Note: AUCg_(0–30′–60′)_ = Area under the curve from salivary cortisol immediately upon awakening, 30 and 60 min later (nmol × h/L); values are means, with standard errors represented by vertical bars.

According to ANOVA, there was no significant difference in the CAR among depressed patients with ELS, without ELS, and controls after placebo (*F* = 0.99; df = 2.0; *p* = 0.38), after dexamethasone (*F* = 1.54; df = 2.0; *p* = 0.23), and after fludrocortisone (*F* = 2.28; df = 2.0; *p* = 0.12). However, upon separately evaluating the CAR in patients with ELS, without ELS, and controls, we found that the effects of dexamethasone and fludrocortisone were different. In controls, we found significant differences in the CAR between placebo and dexamethasone (*p* < 0.001) and between dexamethasone and fludrocortisone (*p* < 0.001), but no difference between placebo and fludrocortisone (*p* = 0.25), indicating suppression of salivary cortisol by GR agonist, but not by MR agonist in controls. In patients without ELS, there were significant differences in the CAR between placebo and dexamethasone (*p* = 0.004). There were no differences between placebo and fludrocortisone (*p* = 0.24) or between dexamethasone and fludrocortisone (*p* = 0.12). These data indicate that, as well as in controls, patients without ELS suppress salivary cortisol only by GR agonist. The situation in depressed patients with ELS was different. There were significant differences in the CAR between placebo and dexamethasone (*p* < 0.001), between placebo and fludrocortisone (*p* = 0.02), and between dexamethasone and fludrocortisone (*p* = 0.001), indicating suppression of salivary cortisol by both GR and MR agonists in patients with ELS (Figure 3).

With respect to the plasma cortisol at 9:00 a.m., there was no significant difference between depressed patients with ELS, without ELS, and controls after placebo (459.7 ± 43.2 vs. 446.2 ± 71.0 vs. 437.0 ± 61.3 nmol/L; *F* = 0.04; df = 2.0; *p* = 0.95), after dexamethasone (54.9 ± 6.5 vs. 101.3 ± 34.4 vs. 77.5 ± 27.1 nmol/L; *F* = 1.10; df = 2.0; *p* = 0.34), and after fludrocortisone (384.7 ± 55.2 vs. 363.0 ± 55.4 vs. 324.2 ± 29.4 nmol/L; *F* = 0.41; df = 2.0; *p* = 0.66). Finally, we correlated the plasma cortisol levels after placebo and CTQ scores in the depressed patients and controls. Interestingly, there was a highly positive correlation between plasma cortisol and the severity of ELS in patients with ELS (*R* = 0.66; *p* = 0.01). No correlation was found in patients without ELS (*R* = −0.54; *p* = 0.20) and in controls (*R* = 0.48; *p* = 0.16).

The objective of the present study was to evaluate the impact of the severity of CTQ scores on, MR and GR in depressive patients with and without ELS for which a multiple regression analysis was conducted. Using CTQ scores as a continuous variable HAM-D_21_, and cortisol measures (CAR after placebo, dexamethasone, fludrocortisone) and BMI we found in a Linear Regression model in depressive patients with ELS: *R* = 0.89; Δ*R*^2^ = 0.79; Δ*F* = 5.31; df = 5; and *p* = 0.025; and in depressive without ELS *R* = 1.0; Δ*R*^2^ = 1.0; Δ*F* = 739.25; df = 5; and *p* = 0.028. In this model, the correlation between CTQ, HAM-D_21_, BMI, CAR (measured as AUC) after placebo, dexamethasone, and fludrocortisone keeps the importance of MR in depressed patients with ELS, but not GR: a significant positive correlation between AUC placebo vs. AUC fludrocortisone (*R* = 0.51; *p* < 0.05); AUC fludrocortisone vs. AUC dexamethasone (*R* = 0.76; *p* < 0.01) and a negative significant correlation between scores of CTQ and BMI (*R* = −0.84; *p* < 0.01). We could not find correlation between AUC placebo vs. AUC dexamethasone (*R* = 0.11; NS) and in the others measures included in the model, see details in Tables 2 and 3.

**Table 2:** **Cortisol awakening response (measured as area under the curve) after placebo, dexamethasone, and fludrocortisone in depressed patients with or without early life stress and controls; and adjusting for CTQ scores, BMI, and HAMD-_21_**.

Mean (SEM)	AUC placebo	AUC dexamethasone	AUC fludrocortisone	*p*	*p* Adjusted
With ELS	36.0 (±4.2)	2.8 (±0.4)*^#^	22.4 (±4.4)^¶^	<0.001	0.025
Without ELS	29.6 (±6.9)	4.5 (±1.6)**	18.4 (±3.8)	0.005	0.028
Controls	40.0 (±3.9)	2.5 (±0.5)^##^	32.3 (±4.4)^¶¶^	<0.001	0.366

*ELS, early life stress; AUC, area under the curve from salivary cortisol immediately upon awakening, 30 and 60 min later (nmol × h/L); SEM, standard error of mean. CTQ, Childhood Trauma Questionnaire; HAM-D_21_, Hamilton Depression Rating Scale. p, Pearson correlation. BMI, body mass index*.

**In depressed patients with ELS**: *p < 0.001 placebo vs. dexamethasone, ^#^*p* = 0.001 dexamethasone vs. fludrocortisone, ^¶^*p* = 0.02 placebo vs. fludrocortisone.

**In patients with depression without ELS**: **p = 0.004 placebo vs. dexamethasone.

***In controls**: *^##^p* < 0.001 placebo vs. dexamethasone, *^¶¶^p* < 0.001 placebo vs. fludrocortisone*.

**Adjusting for CTQ scores, BMI and HAM-D_21_**

***In patients with depression with ELS**: *p* < 0.05 placebo vs. fludrocortisone, *p* < 0.01 fludrocortisone vs. dexamethasone*.

***In patients with depression without ELS**: *p* < 0.05 placebo vs. fludrocortisone, *p* < 0.05 placebo vs. dexamethasone, *p* < 0.01 fludrocortisone vs. dexamethasone*.

**Table 3:** **Pearson correlation between CTQ, HAM-D_21_, BMI, cortisol awakening response (measured as AUC) after placebo, dexamethasone and fludrocortisone in a linear regression model in depressive patients with early life stress (ELS) and without ELS**.

Mean (SEM)	CTQ	AUC placebo	AUC dexamethasone	AUC fludrocortisone	HAMD	BMI
**WITH ELS**						
CTQ	**1**	0.13	−0.34	−0.15	0.43	−0.84**
AUC placebo	0.13	**1**	0.11	0.51*	0.41	−0.03
AUC dexamethasone	−0.34	0.11	**1**	0.76**	−0.31	0.20
AUC fludrocortisone	−0.15	0.51*	0.76**	**1**	−0.05	0.18
HAMD	0.43	0.41	−0.31	−0.05	**1**	−0.40
BMI	−0.84**	−0.03	0.20	0.18	−0.40	**1**
**WITHOUT ELS**						
CTQ	**1**	−0.34	−0.78*	−0.72*	−0.36	0.21
AUC placebo	−0.34	**1**	0.53*	0.69*	−0.35	0.19
AUC dexamethasone	−0.78*	0.53*	**1**	0.70*	−0.26	0.08
AUC fludrocortisone	−0.72*	0.69*	0.70*	**1**	0.15	−0.22
HAMD	−0.36	−0.35	−0.26	0.15	**1**	−0.49
BMI	0.21	0.19	0.08	−0.22	−0.49	**1**

ELS, early life stress; AUC, area under the curve from salivary cortisol immediately upon awakening, 30 and 60 min later (nmol × h/L); CTQ: Childhood Trauma Questionnaire; HAM-D_21_, Hamilton Depression Rating Scale. R, Pearson correlation. BMI, body mass index. *p ≤ 0.05; **p ≤ 0.01.

In patients with depression without ELS, on the other hand we found significant correlation between GR and MR agonists and placebo: a significant positive correlation between AUC placebo vs. AUC fludrocortisone (*R* = 0.69; *p* < 0.05); AUC placebo vs. AUC dexamethasone (*R* = 0.53; *p* < 0.05); AUC fludrocortisone vs. AUC dexamethasone (*R* = 0.70; *p* < 0.01) and a negative significant correlation between CTQ scores vs. AUC dexamethasone (*R* = −0.78; *p* < 0.01) and CTQ scores vs. AUC fludrocortisone (*R* = −0.72; *p* < 0.01), see details in Tables 2 and 3. In healthy controls *R* = 0.80; Δ*R*^2^ = 0.20; Δ*F* = 5.82; df = 5; and *p* = 0.366; a significant positive correlation between AUC placebo vs. AUC fludrocortisone (*R* = 0.69; *p* < 0.05); AUC fludrocortisone vs. AUC dexamethasone (*R* = 0.66; *p* < 0.05).

## Discussion

This study was designed to clarify the status of the impact of ELS in HPA axis response to challenges with GR and MR agonist in depressed patients. We included patients with current depressive episode (Hamilton Rating Scale ≥17) with ELS (65%) and without ELS (35%). Cortisol measures in the saliva and plasma were evaluated after MR or GR agonist. Firstly, we examined the cortisol in depressed patients and healthy controls. Our data demonstrate that in our sample, depressed patients, with high incidence of ELS (65%) and suicide attempts (85%), had significantly lower levels of salivary cortisol compared to control subjects upon waking after placebo.

Our results are consistent with other studies that show low cortisol levels associated with several stress neuropsychiatric disorders, such as posttraumatic stress disorder (PTSD), chronic pain, fibromyalgia/fatigue syndromes, and atypical depression (72–75). Low levels of cortisol have also been demonstrated in depressed trauma survivors (55) and childhood sexual abuse victims (76). In this regard, an important link between trauma and atypical depression comes from studies that exhibit down-regulation of HPA axis due to chronic stress. Some authors have called attention to the role of HPA axis in the etiology of different subtypes of depression. The atypical depression has been associated in some studies with higher rates of neglect/child abuse, family alcohol/drug disorder, high rates of psychiatric comorbidities, and chronicity of depression (77–80). Several studies have also demonstrated a hypoactivity of the HPA axis, a lower activity of CRH, hypocortisolism, and a decrease in activity of afferent noradrenergic pathways in depression with atypical features (73, 81, 82). In contrast, melancholic depression has been associated with a lower incidence of stressful events, lower rates of personality disorders, a lower incidence of suicide attempts, and a hyperactive of the HPA axis (79, 83–85). In this sense, our findings are in line with prior studies, where a pattern of HPA axis hypofunction and reduced secretion of CRH, mediated by an increased negative feedback, appear to be present in depressed patients evaluated in our study (73, 81).

Our results also demonstrate that depressed patients showed a significantly lower CAR after fludrocortisone, but not after dexamethasone compared with healthy controls. These data demonstrate that depressed patients have higher suppression of HPA axis in response to the MR agonist (fludrocortisone), but a similar suppression by GR agonist (dexamethasone), compared to healthy control subjects. Thus, our findings indicate the possibility of an imbalance between GR and MR, with increased MR activity in depressed patients compared with controls.

Although studies of literature have proved the importance of MR in depression, the results about the role of MR in depression are inconsistent. While some studies, ours included, showed increased MR activity, other studies showed that MR function is reduced in depression. MR function can be assessed by MR antagonist (spironolactone), this compound is able to activate the HPA axis blocking MR mediated negative feedback. Young et al. (52) showed a significant increase in cortisol levels in patients treated with spironolactone. Based on these data, the authors suggest that MR activity is increased in patients with depression compared with controls and that the depression is accompanied by a shift in the balance between GR and MR (52). Furthermore, studies have demonstrated in depressed patients an up-regulated MR gene expression in the hypothalamus (86), down-regulation of hippocampal MR in response to antidepressants (87), and reduced residual symptoms in euthymic patients with bipolar disorder (88), suggesting that blocking MR might be promising from a therapeutic perspective. On the other hand, Otte et al. (50) examined the response to antidepressants through stimulation and blockade of MR and found decreased plasma cortisol levels in depressed patients treated with fludrocortisone as adjunct to escitalopram and that the stimulation of MR with fludrocortisone accelerated the response to treatment. Furthermore, the combination of spironolactone and escitalopram increased plasma cortisol levels during treatment (50). There are also studies that suggest that depressed suicide victims showed decreased MR messenger RNA in the hippocampus compared with healthy controls (89). Recently Lembke et al. (49) published a study showing that individuals with PMD compared to healthy control subjects have diminished feedback inhibition of the HPA axis in response to the MR agonist fludrocortisone (49). Our group recently published (60) a study with treatment-resistant depression (TRD) patients showing that TRD had higher cortisol compared with controls after (a) the effect of combined GR/MR stimulation with prednisolone; (b) the effect of prednisolone with the MR antagonist spironolactone; and (c) the effect of spironolactone alone. In healthy controls, spironolactone increased cortisol compared to placebo. The co-administration of spironolactone with prednisolone in controls decreases the suppressive effects of prednisolone. In contrast, in TRD, spironolactone did not increase cortisol compared to placebo and spironolactone with prednisolone had no effect on the suppressive effects of prednisolone. Our data confirmed that TRD is associated with hypercortisolism and these patients no longer show an HPA axis response to the administration of a MR antagonist, suggesting that there is a MR malfunctioning, such as a down-regulation (60). Therefore, these findings suggest that dysregulation of the HPA axis in depression is partially attributable to an imbalance between GR and MR suggesting MR is a promising approach to improve antidepressant treatment in TRD (60).

With regard to GR, there are several studies in the literature with dexamethasone (alone or in combination with CRH) in depression. Most of them have shown an increased activity of the HPA axis in depressive patients compared to healthy controls, associated with hypercortisolemia and reduced inhibitory feedback. These findings suggest that GR function is impaired in major depression, resulting in reduced GR-mediated negative feedback on the HPA axis (34, 41–43, 90, 91). In contrast, in our study, as well as the study of Vreeburg et al. (24) and Gervasoni et al. (92), we did not find cortisol non-suppression by GR agonist (dexamethasone) in the depressed groups. However, most studies that found more non-suppression after dexamethasone among depressed subjects were conducted among more severely depressed patients with melancholic, psychotic, or bipolar depression (43, 91, 93), unlike our sample that consisted predominantly of patients with unipolar depression and ELS. Furthermore, studies have demonstrated that psychotic depression was most clearly associated with prominent non-suppression, whereas the non-suppression rate in non-melancholic was low (73, 74, 81–83, 85).

Concerning the evaluation of impact of ELS in HPA axis response to challenges with GR and MR agonist in depression, our findings indicate that patients with ELS show suppression of salivary cortisol levels after fludrocortisone (MR agonist) and dexamethasone (GR agonist), indicating that patients with ELS are equally sensitive to both GR and MR. In contrast, in depressed patients without ELS and controls, such suppression after fludrocortisone was not found. Patients without ELS and controls showed only suppression by dexamethasone.

However, when we control the data for depression scores (HAMD), BMI and use CTQ scores as continuous measures correlating with CAR AUC (nmol × h/L) after placebo vs. fludrocortisone vs. dexamethasone, the data retain the differences for ELS between after fludrocortisone (MR agonist) but not after dexamethasone (GR agonist), in the same line for depressive patients without ELS the data retain the differences for ELS between after fludrocortisone (MR agonist) but now as well as after dexamethasone (GR agonist). This data may suggest that controlling depression scores, CTQ scores measures the higher severity of childhood trauma and depressive symptoms increase the MR malfunction (60). Thus, our data indicate differences in the functioning of the HPA axis between depressed patients with and without ELS and suggest that patients with ELS are more sensitive to MR agonist than patients without ELS. Therefore, these findings suggest that ELS could be fundamental to impairment of MR function, as found in our study.

Although studies are still restricted, it seems a consensus that ELS is associated with modification of the HPA axis in the first stages of life, which leads to a biological vulnerability to developing depression in adulthood (15, 58, 94). Since the HPA axis is activated in response to stressors, early life stressful events may also have an etiologically significant role in the HPA axis abnormalities found in depression. Increasing evidence indicates that childhood neglect and abuse are risk factors for adult onset depression (14). It has been concluded from these studies that ELS may lead to disruptions in HPA axis functioning and that factors such as age of maltreatment, parental responsiveness, subsequent exposure to stressors, type of ELS, and type of psychopathology or behavioral disturbance displayed may influence the degree and pattern of HPA disturbance (14, 95). Although, there is consensus in the literature that ELS is associated with modification of the HPA axis, the data about the functioning of GR and MR in subjects with ELS are still limited and most studies assess only GR function (8, 55, 57, 58). In this sense, genetics studies in rodents have shown that ELS has epigenomic effects by altering DNA methylation of the GR gene promoter in the hippocampus, leading to functional impairment of the GR and consequently impaired feedback regulation and increased stress responsiveness (96). Still about the role of the ELS in GR, the results of studies with neuroendocrine tests are inconsistent. While, the studies of Heim et al. (8) with abused men with current major depression and Tyrka et al. (57) with healthy adults with parental loss during childhood showed non-suppression by Dex/CRH test, suggesting a decrease of GR activity in subjects with ELS. On the other hand, the study of Newport et al. (55) suggests increased GR activity in women with a history of child abuse and major depression. Moreover, no studies were found in the literature evaluating the role of the ELS specifically in the MR functioning. Thus, because depression is associated with an imbalance between GR and MR (15, 32, 34–36) and based on the data of the literature that demonstrate the influence of ELS in the GR and MR functioning (8, 55, 57). We also conducted separate analyses in depressed patients with and without ELS, in addition to the analysis performed between groups vs. challenge, in order to better investigate our hypothesis that the ELS results in a persistent dysfunction of GR/MR receptors, leading to MR malfunction, in adulthood depressive patient.

Several limitations of the current study should be considered. First, the sample size was relatively small, particularly the subgroup of depressed patients without ELS that reduces the statistical power of our results. Therefore, it is important that our results be interpreted with caution given the sample size. However, despite the limitation of size of our sample and lack of statistical power of our results, according to our knowledge to date, this is the first study published with neuroendocrine challenges that specifically evaluate the functioning of MR in depressive patients with ELS. Second, is the reliance on retrospective self-report questionnaire for investigation of ELS, as the CTQ, used in our study, which is subject to simple forgetting and reporting biases due to mood state of the patient. Third, we did not apply specific instruments to describe our sample with regard to subtypes of melancholic and atypical depression, which could contribute to a better understanding of our neuroendocrine findings. Another potential confounder for our study is that we did not characterize our sample with respect to depressive episodes with or without psychotic features and number of previous depressive episodes, which can influence our biological outcomes. In addition, all our patients were taking antidepressant, which also may have affected the results. Although, this is possible, Kunugi et al. (91) demonstrated that hormonal measures did not differ between patients receiving medication and patients without medication on admission, indicating that medication status did not affect Dex/CRH test results (91). This observation is in line with the finding that the presence or absence of antidepressant treatment and the type and number of antidepressant treatments during the index episode had no effect on hormonal responses to the Dex/CRH test (97). It might also be useful to allow comparison of male and female subjects to ascertain, whether sex steroids and menopausal status can influence HPA axis dysfunction and other hormones like ACTH and aldosterone, the most selective hormone to bind to MR, which could be measured concomitantly to improve the overall assessment of MR sensitivity and function (30). Another limitation in our study is that we evaluated the HPA axis response to challenges with dexamethasone and fludrocortisone and assume that the observed HPA axis suppression is predominantly due to dexamethasone binding at GR, but dexamethasone can also bind to MR and that suppression is predominantly due to fludrocortisone binding at MR, but fludrocortisone can also bind to GR. Indeed, it is possible that fludrocortisone effect might be due in part to minor effects on GR and dexamethasone on MR. Thus, as well as depression needs to be further investigated as to the role of MR receptors in regulating the inhibitory feedback of the HPA axis, changes that ELS generates in the HPA axis need further elucidation (98). Therefore, future studies with larger samples and longitudinal designs to assess the influence of ELS in treatment response with tests that assess both GR and MR, such as prednisolone (a mixed agonist GR/MR), are needed.

## Conclusion

According to our knowledge to date, this is the first study to evaluate HPA axis response to MR stimulation in depressive patients with and without ELS. Our findings indicate that MR activity is increased in depressed patients compared with controls. Furthermore, in spite of the previous limitations described, in depressed patients with ELS, controlling severity of depression, childhood trauma, and BMI there was suppression by fludrocortisone, indicating that patients with ELS are sensitive to MR agonists. In contrast, we find suppression in depressed patients without ELS after both MR and GR agonist. These data could suggest that patients with ELS could be more sensitive to MR agonist than patients without ELS and that ELS could trigger changes in MR activity, but not in GR that might explain the occurrence of distinct results in the subgroups of depression.

However, for better understanding the mechanism by which exposure to ELS leads to such impairment in depression, future studies with larger samples and longitudinal designs ideally should also consider the Environment vs. Gene interaction model. Therefore, once we confirm these data we may develop approaches to early intervention, including new pharmacologic targets and psychoeducational strategies, among others.

## Conflict of Interest Statement

The authors declare that the research was conducted in the absence of any commercial or financial relationships that could be construed as a potential conflict of interest.
